# Geographical distribution of complement receptor type 1 variants and their associated disease risk

**DOI:** 10.1371/journal.pone.0175973

**Published:** 2017-05-17

**Authors:** Thaisa Lucas Sandri, Selorme Adukpo, Dao Phuong Giang, Christian N. Nguetse, Fabiana Antunes Andrade, Hoang van Tong, Nguyen Linh Toan, Le Huu Song, Preetham Elumalai, Kumarasamy Thangaraj, Vijaya Lakshmi Valluri, Francine Ntoumi, Christian G. Meyer, Iara Jose de Messias Reason, Peter G. Kremsner, Thirumalaisamy P. Velavan

**Affiliations:** 1 Institute of Tropical Medicine, University of Tübingen, Tübingen, Germany; 2 Laboratory of Molecular Immunopathology, Federal University of Paraná, Curitiba, Brazil; 3 Noguchi Memorial Institute for Medical Research, University of Ghana, Accra, Ghana; 4 108 Military Central Hospital, Hanoi, Vietnam; 5 Vietnamese - German Center for Medical Research, Hanoi, Vietnam; 6 Vietnam Military Medical University, Hanoi, Vietnam; 7 Kerala University of Fisheries and Oceanic Studies, Kochi, India; 8 CSIR-Centre for Cellular and Molecular Biology, Hyderabad, India; 9 LEPRA- Blue Peter Public Health and Research Center, Hyderabad, India; 10 Fondation Congolaise pour la Recherche Médicale, Brazzaville, Republic of Congo; 11 Duy Tan University, Da Nang, Vietnam; Pennsylvania State University College of Medicine, UNITED STATES

## Abstract

**Background:**

Pathogens exert selective pressure which may lead to substantial changes in host immune responses. The human complement receptor type 1 (CR1) is an innate immune recognition glycoprotein that regulates the activation of the complement pathway and removes opsonized immune complexes. *CR1* genetic variants in exon 29 have been associated with expression levels, C1q or C3b binding and increased susceptibility to several infectious diseases. Five distinct *CR1* nucleotide substitutions determine the Knops blood group phenotypes, namely Kn^a/b^, McC^a/b^, Sl1/Sl2, Sl4/Sl5 and KCAM+/-.

**Methods:**

*CR1* variants were genotyped by direct sequencing in a cohort of 441 healthy individuals from Brazil, Vietnam, India, Republic of Congo and Ghana.

**Results:**

The distribution of the *CR1* alleles, genotypes and haplotypes differed significantly among geographical settings (p≤0.001). *CR1* variants rs17047660*A/G* (McC^a/b^) and rs17047661*A/G* (Sl1/Sl2) were exclusively observed to be polymorphic in African populations compared to the groups from Asia and South-America, strongly suggesting that these two SNPs may be subjected to selection. This is further substantiated by a high linkage disequilibrium between the two variants in the Congolese and Ghanaian populations. A total of nine *CR1* haplotypes were observed. The CR1*AGAATA haplotype was found more frequently among the Brazilian and Vietnamese study groups; the CR1*AGAATG haplotype was frequent in the Indian and Vietnamese populations, while the CR1*AGAGTG haplotype was frequent among Congolese and Ghanaian individuals.

**Conclusion:**

The African populations included in this study might have a selective advantage conferred to immune genes involved in pathogen recognition and signaling, possibly contributing to disease susceptibility or resistance.

## Introduction

Complement receptor type 1 (CR1) is widely recognized to play a role in disease pathophysiology, diagnosis, prognosis and in therapy [1]. The gene encoding human CR1 is located on chromosome 1 (1q32.2; OMIM 120620) [2–4]. CR1 belongs to the regulator of complement activation family (RCA) and is a transmembrane glycoprotein (single chain type 1), which occurs either in membrane-bound or soluble forms [2,5]. CR1 is predominantly involved in the transport of circulating immune complexes to the reticuloendothelial system.

CR1 acts as a regulator in the three pathways of the complement system [2], namely the classical, the lectin and the alternative pathway. It enhances phagocytosis of opsonized particles together with the complement components C3b, C4b, C1q, mannose-binding lectin and ficolin-2, thereby facilitating clearance of opsonized immune complexes. In the presence of Factor I, CR1 suppresses the complement cascade by inactivating C3b and C4b [6]. CR1 comprises of 30 short complement regulator (SCR) domains, known as complement control protein repeats (CCPs). Four protein isoforms have been identified based on their molecular weight and the number of *CR1* exons [3]. Groups of seven CCPs are organized into four long homologous repeats (LHRs A to D) [7,8].

CR1 is also expressed on cells involved in both innate and adaptive immune responses [9–11]. The erythrocyte CR1 binds to circulating immune complexes and to complement-coated particles to transport them to the liver or spleen for subsequent phagocytosis [2,3]. CR1 deficient mice showed decreased and delayed IgM and IgG responses to West-Nile virus, thus increasing mortality [12]. Moreover, *in vitro* studies have shown that CR1 has distinct adjuvant properties [13–16], probably due to its involvement in uptake of antigen by antigen-presenting cells [17].

Three types of polymorphisms have been characterized in the *CR1* gene, namely those generating size variants, those resulting in copy number differences on red blood cells and polymorphisms forming the Knops blood group antigens [1,18]. Five distinct *CR1* nucleotide substitutions determine the Knops blood group phenotypes: Knops (rs41274768, Kn^a/b^, p.N1540S), McCoy (rs17047660, McC^a/b^, p.K1590E), Swain-Langley/Villien (rs17047661, Sl1/Sl2, p.R1601G), Swain-Langley (rs4844609, Sl4/Sl5, p.T1610S), and the KCAM antigens (rs6691117, KCAM+/-, p.I1615V) [19–23].

In the process of pathogen evasion from the host´s immune system, pathogens bind to complement receptors and other regulatory proteins to facilitate their uptake by host cells. This may considerably downregulate and impair the function of the complement system [24]. For instance, CR1 has been reported to facilitate entry of intracellular pathogens into host cells and CR1 protein levels are associated with disease susceptibility. Among protozoan parasites, CR1 mediates immune adherence of intracellular *Leishmania* amastigotes [25] to present them to macrophages, the preferred habitat of *Leishmania* [26,27]. Low CR1 levels were associated with a decreased degree of opsonisation in patients with chronic *Trypanosoma cruzi* infection [28]. Among viral infections, CR1 has been shown to be a secondary receptor for Epstein-Barr virus (EBV) [29] and to expedite the entry of EBV into cells [30,31]. CR1 is associated with the pathogenesis caused by SARS-CoV [32], adenoviruses [33] and other viral infections such as HIV and HCV [30].

The present study utilized samples from five populations originating from Brazil, Ghana, Republic of Congo, India and Vietnam and aimed to assess the distribution of the different Knops blood group antigens and functional *CR1* genetic variants [rs17259045, rs41274768 (Kn^a/b^), rs17047660 (McC^a/b^), rs17047661 (Sl1/Sl2), rs4844609 (Sl4/Sl5), rs6691117 (KCAM+/-)] in exon 29 that were involved in pathogen recognition and signaling, possibly contributing to disease susceptibility or resistance.

## Methods

### Ethics statement

The study was approved by the Ethics Committee of the Hospital de Clínicas in Curitiba, Brazil, the institutional Review Board of the Tran Hung Dao Hospital, Hanoi, Vietnam, the Ethics Committee of the CSIR-Centre for Cellular and Molecular Biology, Hyderabad, India, Ethics Committee of the LEPRA-Blue Peter Public Health and Research Centre; the Ethics Committee of the Fondation Congolaise pour la Recherche Médicale, Brazzaville, Republic of Congo and the Ethics Committee of the Noguchi Memorial Institute for Medical Research, Ghana. Informed written consent was received from all studied participants (consent from parents if the participant was under 18 years old).

### Study population

A total of 441 DNA samples from healthy individuals were utilized. Investigations were carried out in populations from Brazil [n = 102; mean age 51±7; 48% (49/102) were female and 52% (53/102) male], Ghana [n = 77; mean age 5±3; 45% (28/62) were female and 55% (34/62) male], Republic of Congo [n = 77; mean age 3±3; 49% (38/77) were female and 51% (39/77) male], India [n = 86; mean age 32±18; 39% (30/78) were female and 61% (48/78) male] and Vietnam [n = 99; mean age 26±5; 40% (36/89) were female and 60% (53/89) male].

### CR1 genotyping

In order to assess the distribution of six functional variants [rs17259045, rs41274768 (Kn^a/b^), rs17047660 (McC^a/b^), rs17047661 (Sl1/Sl2), rs4844609 (Sl4/Sl5), rs6691117 (KCAM+/-)], the complete *CR1* exon 29 including their intron-exon boundaries was screened by direct sequencing in the 441 DNA samples (Table 1). A fragment of 884 bp in exon 29 of the *CR1* gene was amplified by polymerase chain reaction (PCR) using the *CR1* locus specific primer CR1F (5'-TCT TCA TAA ATA ATG CCA GAA GTG G-3') and CR1R (5'-TGC CAA TTT CAT AGT CCT TAT ACA C-3'). PCR amplifications were carried out in a 25 μl volume of reaction mixture containing 10X PCR buffer, 3.0 mM MgCl2, 0.2 mM dNTPs, 0.2 μM of each primer, 1 unit of Taq polymerase (Qiagen GmbH, Hilden, Germany) and 20 ng of genomic DNA on a TProfessional Basic Thermocycler (Biometra GmbH, Göttingen, Germany). Cycling parameters were initial denaturation at 94°C for 5 minutes followed by 40 cycles of denaturation at 94°C for 30 seconds, annealing at 55°C for 30 seconds and elongation at 72°C for 1 minute, and a final elongation step at 72°C for 10 minutes. PCR fragments were stained with SYBR Safe DNA Gel Stain (Invitrogen, Carlsbad, USA) and visualized on 1.5% agarose gels. PCR products were subsequently purified using Exo-SAP-IT (USB, Affymetrix, Santa Clara, CA, USA) and the purified products were directly used as templates for sequencing using the BigDye terminator v. 1.1 cycle sequencing kit (Applied Biosystems, Foster City, CA, USA) on an ABI 3130XL DNA sequencer according to the manufacturer’s instructions. DNA polymorphisms were identified by assembling the sequences with the reference sequence of the *CR1* (NM_000573) using Geneious v9.1.4 software (Biomatters Ltd, Auckland, New Zealand) and reconfirmed visually from their respective electropherograms.

**Table 1:** Genotypes and allele frequencies of the investigated six *CR1* variants among world populations.

*CR1* SNPs		Brazilian n = 102 (%)	Vietnamese n = 99 (%)	Indian n = 86 (%)	Congolese n = 77 (%)	Ghanaian n = 77 (%)	*p* value
**rs17259045*A/G***	*AA*	82 (80)	99 (100)	84 (98)	77 (100)	77 (100)	< 0.002
	*AG*	20 (20)	0	2 (2)	0	0	
	*GG*	0	0	0	0	0	
	*A*	184 (90)	198 (100)	170 (99)	154 (100)	154 (100)	
	*G*	20 (10)	0	2 (1)	0	0	
**rs41274768*G/A***	*GG*	98 (96)	99 (100)	85 (99)	77 (100)	77 (100)	NA
	*GA*	4 (4)	0	1 (1)	0	0	
	*AA*	0	0	0	0	0	
	*G*	200 (98)	198 (100)	171 (99)	154 (100)	154 (100)	
	*A*	4(2)	0	1 (1)	0	0	
**rs17047660*A/G***	*AA*	99 (98)	99 (100)	86 (100)	46 (60)	35 (45)	<0.0001
	*AG*	2 (2)	0	0	27 (35)	34 (44)	
	*GG*	0	0	0	4 (5)	8 (10)	
	*A*	200 (99)	198 (100)	172 (100)	119 (77)	104 (67)	
	*G*	2 (1)	0	0	35 (23)	50 (33)	
**rs17047661*A/G***	*AA*	94 (93)	99 (100)	86 (100)	7 (9)	7 (9)	<0.01
	*AG*	7 (7)	0	0	31 (40)	28 (36)	
	*GG*	0	0	0	39 (51)	42 (55)	
	*A*	195 (96)	198 (100)	172 (100)	45 (29)	42 (26)	
	*G*	7 (4)	0	0	109 (71)	112 (74)	
**rs4844609*T/A***	*TT*	99 (98)	99 (100)	86 (100)	77 (100)	77 (100)	NS
	*TA*	2 (2)	0	0	0	0	
	*AA*	0	0	0	0	0	
	*T*	200 (99)	198 (100)	172 (100)	154 (100)	154 (100)	
	*A*	2 (1)	0	0	0	0	
**rs6691117*A/G***	*AA*	61 (60)	37 (37)	21 (24)	0	3 (4)	< 0.006
	*AG*	33 (33)	53 (53)	39 (46)	19 (25)	10 (13)	
	*GG*	7 (7)	9 (9)	26 (30)	58 (75)	64 (83)	
	*A*	155 (77)	127 (64)	81 (47)	19 (12)	16 (9)	
	*G*	47 (23)	71 (36)	91 (53)	135 (88)	138 (91)	

NS, not significant; NA, not applicable

### Statistical analysis

Statistical analyses were performed using the GraphPad Prism 3.0 software package (GraphPad Software, La Jolla, CA, USA) and Stata 12.0 (StataCorp, College Station, TX, USA). Normal Chi square and two tailed Fisher’s exact tests were calculated to determine the differences of genotype, allele and haplotype frequencies among the different ethnicities. Genotype and allele frequencies were determined by simple gene counting and haplotypes were reconstructed by using the expectation-maximum (EM) algorithm as implemented in the Arlequin v3.5.2.2 software (http://cmpg.unibe.ch/software/arlequin35/Arl35Downloads.html). The significance of deviations from Hardy Weinberg equilibrium was tested using the approach of Guo and Thompson random-permutation procedure implemented in Arlequin v. 3.5.2.2 software. Linkage disequilibrium (LD) analysis was performed using the Haploview v. 3.2 program (https://www.broadinstitute.org/haploview/downloads). The level of significance was set to a p-value of <0.05.

## Results

The frequencies of *CR1* genotypes in the five populations were in Hardy Weinberg equilibrium (p>0.05). The allele and genotype frequencies of the *CR1* SNPs rs17259045, rs17047660 (McC^a/b^), rs17047661 (Sl1/Sl2) and rs6691117 (KCAM+/-) differed significantly among the groups (p≤0.01) (Table 1). Genotype frequencies of the *CR1* variants rs41274768 (Kn^a/b^) and rs4844609 (Sl4/Sl5) did not differ. The rs17259045*AG* genotype and the rs17259045*G* allele were more frequent in the Brazilian population. Moreover, the G carriers (*AG* and *GG*) and the *G* allele of variants rs17047660 (McC^a/b^), rs17047661 (Sl1/Sl2) and rs6691117 (KCAM+/-) were observed more commonly among the two African populations (Republic of Congo, Ghana). Interestingly, among Congolese and Ghanaian individuals the minor allele of SNPs rs17259045*A/G*, rs41274768*G/A* (Kn^a/b^) and rs4844609*T/A* (Sl4/Sl5) did not occur at all; this allele was observed exclusively in Brazilian individuals. Except for rs6691117 (KCAM+/-), the Vietnamese population was monomorphic. The Indian group was monomorphic for three of the SNPs, but not for rs17259045, rs41274768 (Kn^a/b^) and rs6691117 (KCAM+/-). Brazilian individuals were polymorphic for all SNPs (Table 1). The Knops blood antigen distribution among the studied populations is summarized in Table 2.

**Table 2:** Knops blood group antigens distribution among world populations.

*CR1* variants	Amino acid substitution	Knops blood antigens	Brazil n = 202 (%)	Vietnam n = 198 (%)	India n = 172 (%)	Congo n = 154 (%)	Ghana n = 154 (%)	*p* value
**rs41274768**	V1561M	Kn^a^	200 (98)	198 (100)	171 (99.4)	154 (100)	154 (100)	NS
		Kn^b^	4 (2)	0	1 (0.6)	0	0	
**rs17047660**	K1590E	McC^a^	200 (99)	198 (100)	172 (100)	119 (77.3)	104 (67.5)	<0.0001
		McC^b^	2 (1)	0	0	35 (22.7)	50 (32.4)	
**rs17047661**	R1601G	Sl1	195 (96.5)	198 (100)	172 (100)	45 (29.2)	42 (27.3)	<0.0001
		Sl2	7 (3.5)	0	0	109 (70.8)	112 (72.7)	
**rs4844609**	T1610S	Sl4	200 (99)	198 (100)	172 (100)	154 (100)	154 (100)	NA
		Sl5	2 (1)	0	0	0	0	
**rs6691117**	I1615V	KCAM+	155 (76.7)	127 (64.1)	81 (47.1)	19 (12.3)	16 (10.4)	<0.0001
		KCAM-	47 (23.3)	71 (35.9)	91 (52.9)	135 (87.7)	138 (89.6)	

NS, not significant; NA, not applicable

**Fig 1:**
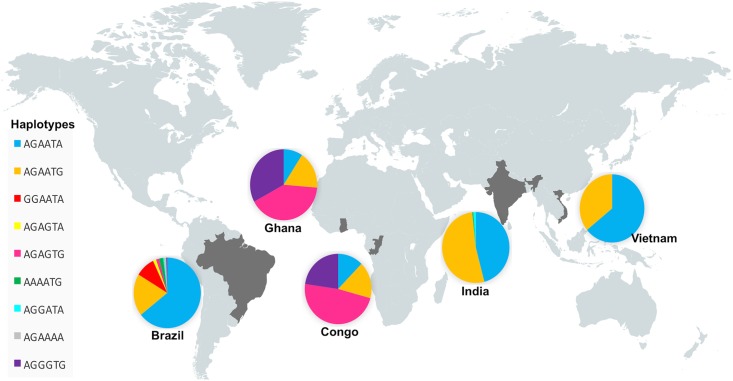
Distribution of *CR1* haplotypes in world populations.

**Fig 2:**
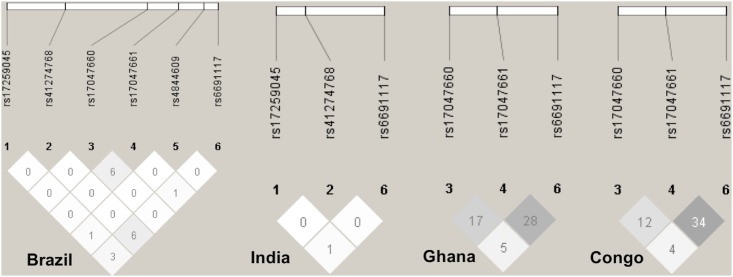
Linkage disequilibrium (LD) of *CR1* single nucleotide polymorphisms. LD was calculated based on the data for Brazilian, Indian, Congolese and Ghanaian populations, being the pairwise correlation coefficient values (r2) between tag SNPs referred by numbers inside the squares that show the amount of LD between two SNPs. Black, gray, and white squares represent high, medium and low levels of LD, respectively. Relative position of SNPs on *CR1* gene is indicated on the abscissas. (*) Vietnamese population was found monomorphic for five variants except for the variant rs6691117 in *CR1* gene, therefore the LD plot for Vietnamese population was not possible.

Haplotypes were reconstructed from the six *CR1* variants. A total of nine haplotypes were observed. The haplotype distributions are summarized in Table 3 and Fig 1. The CR1*AGAATA haplotype was more frequent among the Brazilian and Vietnamese populations; CR1*AGAATG occurred frequently among the Indian and Vietnamese groups, while CR1*AGAGTG was observed frequently among Congolese and Ghanaian individuals. The CR1*AGGGTG and CR1*AGAGTG haplotypes were observed only in Brazil and Africa, being far more frequent among the Congolese and Ghanaian groups. Interestingly, CR1*GGAATA was exclusively observed in the Brazilian population. Linkage disequilibrium (LD) analysis between SNPs revealed medium levels of LD for SNPs rs17047661 (Sl1/Sl2) and rs6691117 (KCAM+/-) and for rs17047660 (McC^a/b^) and rs17047661 (Sl1/Sl2) in the Congolese and Ghanaian study groups (Fig 2).

**Table 3:** Reconstructed *CR1* haplotype distribution among world populations.

*CR1* haplotypes (+4659/+4721/+4808/+4841/+4868/+4883)	Brazil n = 202 (%)	Vietnam n = 198 (%)	India n = 172 (%)	Congo n = 154 (%)	Ghana n = 154 (%)	*p* value
CR1*AGAATA	130 (64)	127 (64)	79 (45.9)	19 (12.3)	14 (9)	<0.0005
CR1*AGAATG	39 (19)	71 (36)	90 (52.3)	26 (17)	26 (16)	<0.0016
CR1*GGAATA	19 (9)	0	0	0	0	NA
CR1*AGGGTG	1 (0.5)	0	0	35 (22.7)	51 (33)	<0.0001
CR1*AGAGTG	3 (1.5)	0	0	74 (48)	63 (41)	<0.0001
CR1*AAAATG	4 (2)	0	1 (0.6)	0	0	NS
CR1*AGGATA	1 (0.5)	0	2 (1.2)	0	0	NS
CR1*AGAAAA	2 (1)	0	0	0	0	NA
CR1*AGAGTA	3 (1.5)	0	0	0	0	NA

NS, not significant; NA, not applicable

## Discussion

Pathogens exert strong selective pressure on the human host, leading to substantial changes in host immune regulation thereby evading immune responses. This study utilized samples from population exposed to diverse infectious diseases, where a strong selective pressure is exerted by these infectious pathogens on the human immune locus. The samples utilized in this study are from different case-control cohorts investigated for possible associations of *CR1* variants with different infectious diseases (unpublished data). Brazilian, Vietnamese and Indian samples utilized in this study are from an endemic area to Chagas disease, viral hepatitis and leprosy respectively. The Republic of Congo and Ghanaian samples are from malaria holoendemic sites.

*CR1* genetic variants in exon 29 are associated with CR1 expression levels, C1q or C3b binding activity and increased susceptibility to various infectious diseases. This study investigated the entire exon 29 of *CR1* in five diverse populations in order to assess the distribution of Knops blood group antigens and the distinct functional *CR1* SNPs. Such studies on geographically diverse populations can provide insights on how these *CR1* alleles have spread in populations and contribute to the understanding of natural selection.

Allele and genotype frequencies of *CR1* variants in exon 29 [rs17259045, rs41274768 (Kn^a/b^), rs17047660 (McC^a/b^), rs17047661 (Sl1/Sl2), rs4844609 (Sl4/Sl5), rs6691117 (KCAM+/-)] as well as their haplotype frequencies were differently distributed among the Brazilian, Vietnamese, Indian, Congolese and Ghanaian study groups. So far, the frequencies of these variants and especially, the distribution of blood group antigens have not been described explicitly for central African populations yet.

*CR1* variants rs17047660*A/G* (McC^a/b^) and rs17047661*A/G* (Sl1/Sl2) were observed to be polymorphic only in the African groups compared to those from Asia and Brazil, indicating that the frequencies of these two SNPs result from a strong selective bias exerted by exposure to distinct pathogens especially by *Plasmodium falciparum*. This is substantiated by a high linkage disequilibrium between the two variants. Of the reconstructed *CR1* haplotypes, CR1*AGAGTG and CR1 *AGGGTG were observed to be unique among the Congolese and Ghanaian groups. CR1*AGAGTG contains the allele of the rs17047660*A*. This locus also determines the Knops blood group antigen McC^a/b.^ Studies have demonstrated that this blood group antigen is dominant among many ethnic groups of African ancestry living in malaria endemic regions [34].

Higher rates of adaptive evolution are expected to occur especially in genes involved in the immune system, as these gene loci coevolve with pathogens. This is largely contributed by two factors the genetics of the population and natural selection. Immune genes tend to evolve rapidly as selection pressure is changing continuously due to various pathogenic challenges. Therefore, positive selection of rs17047660*A/G* (McC^a/b^) and rs1704661*A/G* (Sl1/Sl2) loci is expected in sub-Saharan African populations exposed to distinct pathogenic challenges (e.g. falciparum malaria). Such a selective advantage occurs mainly in immune genes involved in pathogen recognition and signaling, and the *CR1* is one of such genes involved in innate immunity.

In addition, the reported frequencies of these two loci, rs17047660*A/G* (Sl4/Sl5*)* and rs1704661*A/G* (Sl1/Sl2), in this study were in accordance with frequencies observed in other East and West African ethnicities as reported in the 1000 Genomes database (https://www.ncbi.nlm.nih.gov/variation/tools/1000genomes). The frequencies in other African populations correspond to the frequencies observed in this study [rs17047660*A/G* (McC^a/b^): Gambian 0.67/0.32, Kenyan 0.69/0.31, Sierra Leone 0.71/0.29 and Yoruba 0.73/0.27; whereas for rs17047661*A/G* (Sl1/Sl2): Gambian 0.21/0.78, Kenyan 0.30/0.70, Sierra Leone 0.21/0.79 and Yoruba 0.30/0.70]. Also the reported frequencies in other studied Asian and Brazilian populations were in accordance with the frequencies described in the 1000 Genomes database.

There is growing evidence of ethnic differences in susceptibility to some infectious diseases and of genetic adaptation to diverse pathogens [18,35]. This study investigated five antigens of the Knops blood group including the Knops (rs41274768, Kn^a/b^, p.N1540S), the McCoy (rs17047660, McC^a/b^, p.K1590E), the Swain-Langley/Villien (rs17047661, Sl1/Sl2, p.R1601G), the Swain-Langley (rs4844609, Sl4/Sl5, p.T1610S), and the KCAM antigens (rs6691117, KCAM+/-, p.I1615V) [19–23]. These Knops blood group polymorphisms have been found associated with various infectious diseases (Table 4). In particular, the two Knops blood group variants McC^b^ (rs1704660*G*, E1590K) and Sl2 (rs1704661*G*, R1601G) have specific distributions among African populations, which has been related to selective pressure by malaria in Africa [36–42]. The substitution of lysine to glutamic acid at 1590 aa position modulates the epitope conformation and serologic reactivity due to its surface exposed feature, affecting the overall CR1 binding capacity [22]. A high frequency of the rs1704661*G* (Sl2) allele was observed in the African groups. The high frequency of the rs6691117*G* (KCAM-, I1615V) allele in Africa and India indicates that this allele, similar as the rs1704660*G* (McC^b^) and rs1704661*G* (Sl2) alleles, might also be subjected to selection. The presence of rs1704661*G* (McC^b^), which is almost limited to African populations, suggests that rs1704661*A* (Sl1) may be the ancestral allele [43]. Also a differential distribution of rs6691117*A/G* (KCAM+/-) variants was observed. For instance, in the Vietnamese and Brazilian groups, rs6691117*A* (KCAM+) is a major allele, while the variant rs6691117*G* (KCAM-) was observed to be the major allele in Africa. A study from India compared exon 29 *CR1* variants in endemic and non-endemic populations and concluded that a differential association with falciparum malaria in regions of varying disease endemicity exists [44]. However, the Indian samples from the present study originate from an area not endemic for malaria.

**Table 4:** Significance of *CR1 exon 29* Single nucleotide polymorphisms.

*CR1* genetic variants	Knops antigens	Amino acid change	Associated outcome	Reference(s)
rs17259045 (4659A>G)		N1540S	Alzheimer disease	[45]
rs41274768 (4721G>A)	Kn^a/b^	V1561M	Sickle cell trait	[38]
rs17047660 (4808A>G)	McC^a/b^	K1590E	Sickle cell trait	[38]
			Malaria	[36,37,40,42]
			Tuberculosis	[46]
			Leprosy	[47]
rs17047661 (4841A>G)	Sl1/Sl2	R1601G	Sickle cell trait	[38]
			Malaria	[36,37,39,41,42,48]
			Tuberculosis	[46]
rs4844609 (4868T>A)	Sl4/Sl5	T1610S	Alzheimer disease	[49–52]
			Cognitive decline	[53,54]
rs6691117 (4883A>G)	KCAM +/-	I1615V	Erythrocyte Sedimentation Rate	[55]
			Alzheimer Disease	[56]
			Gastric cancer	[57]
			Lung cancer	[58]
			Glioblastoma multiforme	[59]
			Preterm birth	[60]

Taken together, this study revealed significant differences in allele, genotype and haplotype frequencies of *CR1* SNPs in five populations. A limitation of this study might be a small sample size. However, this study, first to include population from Central Africa, may provide an increased understanding of the contribution of red blood cell phenotypes and the complement regulator protein with regard to possible associations with infectious diseases. Further studies are warranted with increased sample sizes, to determine the role of CR1 in disease associations and pathogenesis mechanisms.
